# Machine Learning and Vision: Advancing the Frontiers of Diabetic Cataract Management

**DOI:** 10.7759/cureus.66600

**Published:** 2024-08-10

**Authors:** Najah K Mohammad, Ibrahim A Rajab, Rania H Al-Taie, Mustafa Ismail

**Affiliations:** 1 Department of Ophthalmology, University of Baghdad, Baghdad, IRQ; 2 Department of Surgery, Mustansiriyah University, Baghdad, IRQ; 3 Department of Surgery, University of Baghdad, Baghdad, IRQ

**Keywords:** interdisciplinary collaboration, computational predictions, aldose reductase inhibitors, diabetic cataracts, machine learning

## Abstract

This comprehensive review explores the integration of machine learning (ML) in managing diabetic cataracts. It discusses the potential application of ML to identify novel methodologies for early detection, diagnosis, and therapeutic interventions. The review also addresses clinical translation challenges, including pharmacokinetics properties and ethical considerations. The approach toward cataractogenesis, therefore, has to be from a holistic viewpoint, bringing oxidative stress and metabolic disturbances to the top of importance. It outlines the important requirements, including continued research, diversified datasets, and uses interdisciplinary collaborations in making improvements in ML models and thereafter bridging the gap between computational promise and clinical implication, with the aim to help in the maximization of patient care in the management of diabetic cataract. A literature search through databases like PubMed and Scopus focusing on understanding of current innovations, challenges, and future directions in employing ML in diabetic cataract management was undertaken. This review has explored both recent and foundational studies in order to explain the development and gaps of current research with an aim to enhance outcomes of patient care by promoting future investigation.

Key findings revealed a wide application of ML in ophthalmology including treatment identification, cataract detection and grading, and improving the surgical outcomes. However, this is accompanied by some obstacles, including risk of bias, concerns regarding artificial intelligence application as a diagnostic tool, and legal regulations. ML promises extraordinary developments in the treatment of diabetic cataracts through betterment in diagnosis, treatment, and patient care. With this, it is full of clinical translation and ethical challenges, yet there is recognition in general that continuous model refinement and interdisciplinary collaboration, along with the expansion of the two identified key elements in enhancing patient outcomes, are essential for this to continue.

## Introduction and background

Merging machine learning (ML) and ophthalmology is a new frontier in advancing the field of ophthalmology. The combination harbors a new realm for the management of diabetic cataracts using new methodologies for early detection, diagnosis, and therapeutic interventions. In this paper, we highlight a comprehensive review of the application of AI to the schemes of management of diabetic cataracts in ophthalmic diseases. The integration of ML into ophthalmic research and clinical practice has shown promise regarding diagnostic accuracy, optimizing therapeutic outcomes, and tailoring individualized management plans. However, from the theoretical, computational model to actual clinical implementation, this path is fronted with different challenges, including ethical considerations, data security, and cross-disciplinary collaboration. It is important to note that cataracts are a major cause of visual impairment worldwide, contributing up to 18.4% of its cases and 33.4% of blindness causes [1]. A fact that made it a critical public health challenge. The conversion of glucose into sorbitol within the human eye's lens, a process involved in diabetic cataract formation, is regulated by the aldose reductase (AR) enzyme. Thus, research has demonstrated that aldose reductase inhibitors (ARIs) could significantly reduce the incidence of diabetic cataracts [2]. This review attempts to advocate for agreeable integration of ML in cataract management, mainly through access to the latest literature. It clearly outlines the need for continued innovation, rigorous validation, and ethical vigilance in shrinking the gap between AI and clinical reality, aiming to improve outcomes for patients with diabetic cataracts.

## Review

Method

**Figure 1:**
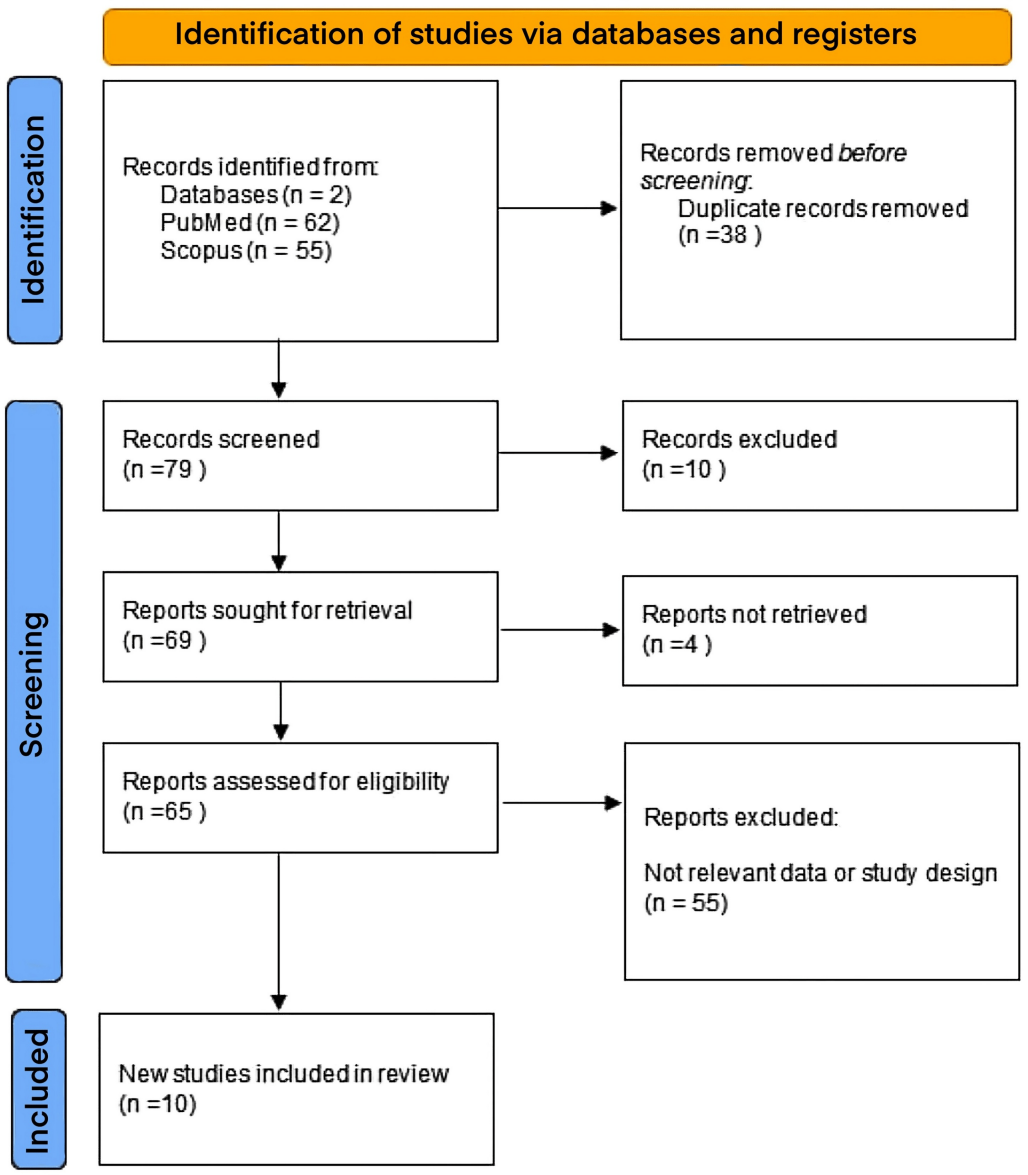
PRISMA 2020 flow diagram for new systematic reviews which included searches of databases and registers only PRISMA - Preferred Reporting Items for Systematic Reviews and Meta-Analysis

The methodology, which integrated ML in the management of diabetic cataracts and, at large, ophthalmology in our comprehensive review, was systematic during the compilation of the literature and analysis and was performed using the Preferred Reporting Items for Systematic Reviews and Meta-Analysis (PRISMA) guidelines [3] (Figure 1). Initially, we identified similar and recent articles through comprehensive database searches, including PubMed and Scopus, using keywords related to "machine learning", "diabetic cataracts", and "ophthalmology". We prioritized articles published within the last ten years to ensure relevance and recency but also included foundational studies to provide historical context. Further, we applied inclusion criteria focusing on studies that demonstrated the application of ML in diagnosing, treating, or managing diabetic cataracts and related ophthalmic conditions. Exclusion criteria removed articles not directly related to ML's role in clinical or therapeutic advancements in diabetic cataracts. Each selected article underwent a qualitative assessment to evaluate its contribution to the field, focusing on methodology, results, and conclusions. The risk of bias assessment is calculated using the PROBAST assessment tool (Table 1) [4]. 

**Table 1:** Risk of bias assessment using the PROBAST tool

Study	Participant selection	Predictors	Outcome	Analysis
Chen et al. (2023) [5]	Low risk	Low risk	Low risk	Low risk
Crane et al. (2023) [6]	Unclear risk	Low risk	High risk	Low risk
Gao et al. (2023) [7]	Unclear risk	Low risk	High risk	Low risk
Gunasekeran et al. (2023) [8]	High risk	Low risk	Low risk	Low risk
Hecht et al. (2018) [9]	Low risk	Low risk	Low risk	Low risk
Lacombe et al. (2023) [10]	Unclear risk	Low risk	High risk	Low risk
Li et al. (2024) [11]	Low risk	Low risk	Low risk	Low risk
Nusinovici et al. (2020) [12]	Low risk	Low risk	High risk	Low risk
Yang et al. (2022) [13]	Low risk	Low risk	Low risk	Low risk
Young et al. (2021) [14]	Unclear risk	Low risk	High risk	Low risk

Results

The results of this comprehensive review, drawing from the latest studies and articles, reveal a multifaceted landscape of ML applications in ophthalmology (Table 2) [5-14].

**Table 2:** Comprehensive overview of machine learning application in diabetic cataract management SMO - sequential minimal optimization; ML - machine learning; AR - aldose reductase; EHR - electronic health record; AI - artificial intelligence; AUC - area under the curve; SVM - support vector machine; SD-OCT - spectral domain optical coherence tomography; DR - diabetic retinopathy; ARC - age‐related cataracts; CDM - cataracts with diabetes mellitus; CHM - cataracts with high myopia

Study ID	Author	Year	Country	Sample size	Study design	Population Characteristics	Detailed ML techniques	Comparison methods	Outcomes	Performance metrics	Results	Quality assessment	Limitations
1	Chen et al. [5]	2023	USA	Not stated	Computational predictive modeling study	Patients with diabetic cataracts	Multilayer Perceptron, LogitBoost, SMO, MultiClassClassifier	None	High accuracy of ML models in predicting AR inhibitors	Cross-validation accuracy of 90%; high correlation with binding free energy in computational docking	Computational docking testing of the predicted inhibitors gave a high level of correlation between the ML prediction score and binding free energy	High	The experiment is limited by a lack of experimental data and aldose reductase inhibitors. Despite being a well-studied protein, public data is scarce, and few drugs have been tested. The ML models also exhibit biases because frequently studied inhibitor classes dominate the models, a known issue in supervised learning methods.
2	Crane et al. [6]	2023	USA	3662	Experimental study	Patients from Asia Pacific with diabetic retinopathy and simulated cataract	Convolutional neural network (CNN) with transfer learning	AI accuracy applied to color fundus photos (CFPs) with simulated cataracts (SCs) in detecting diabetic retinopathy (DR)	SCs severity significantly impaired AI accuracy.	Accuracy decreased from 97% without SC to 55% with severe SC; sensitivity and specificity also decreased with SC	Accuracy decrease with SC	High	limitations in simulating real cataract effects and generalizability of results
3	Gao et al. [7]	2023	USA	800,000	Multiphase study that combines elements of computational phase and retrospective cohort study phase	Cataract patients from the TriNetX platform	KG-Predict	Random compounds vs. ML-predicted compounds	Binding energies, cross-validation accuracy	High correlation between ML prediction score and binding free energy; ML models accurately predicted AR inhibitors	High correlation with binding free energy	High	Using an evolving set of 12 genes for diabetic cataracts and the EHR database's lack of detailed drug usage information, such as duration, dosage, and patient compliance. The AI system can adapt to new data.
4	Gunasekeran et al. [8]	2022	Singapore	1176	Cross-sectional survey study	Ophthalmologists from different nations	Not specific	Anonymous web-based electronic survey	Diagnostic accuracy, sensitivity, specificity, acceptability	The survey was iteratively refined through literature review to develop semi-structured dichotomous and Likert questions	Assistive tools were the most acceptable form of clinical AI application in ophthalmology (89.2%). Diagnostic tools intended for use by ophthalmologists received the lowest acceptance (59.1%)	High	Majority from Asia Pacific, less from the West. Snowball sampling excluded primary care providers (PCPs). Potential bias towards stakeholders actively engaged in professional associations. Use of logistic regression assumes linear relationships, which may not account for non-linear relationships.
5	Hecht et al. [9]	2019	Israel	153	Diagnostic accuracy study	Patients with diabetic macular edema or pseudophakic cystoid macular edema	Advanced ML algorithms (Logistic regression, SVM, decision trees, random forest, gradient-boosted trees)	Human grading and comparison of different algorithms	Diagnostic accuracy, sensitivity, specificity	Sensitivity: 94%-98%, Specificity: 94%-95%, AUC: 0.937-0.987 depending on method	Using only 3 to 6 spectral domain OCT parameters, they achieved a sensitivity of 94% to 98%, specificity of 94% to 95%, and an area under the curve of 0.937 to 0.987 (depending on the method) for confirming a diabetic etiology. While a simple decision flowchart achieved a sensitivity of 96%, a specificity of 95%, and an area under the curve of 0.937.	High	Combining datasets from different sources may induce biases due to differing SD-OCT image acquisition protocols. The small number of "mixed" cases led to misclassification and reduced the classifier's diagnostic potential. The study did not evaluate the impact of the classifier on patient management or clinical outcomes.
6	Lacombe et al. [10]	2015	France	96	Proof-of-concept study	healthy (n = 47), diabetic (n = 19), and galactosemic patients (n = 30)	support vector machine leave-one-out cross-validation (SVM-LOOCV) classifier	Traditional diagnostic methods	Diagnostic accuracy, sensitivity, specificity	High accuracy (91%), sensitivity (89%), specificity (90%) for cataract detection	High accuracy, sensitivity, specificity	High	This is a proof-of-concept study which needs to be confirmed on a larger population
7	Li et al. [11]	2024	China	1578	Prospective study	Patients with various blindness-causing eye diseases. Aged 18 years or older.	Decision-analytic Markov model	Ophthalmologists' grading	Accuracy of ML models	High accuracy for diabetic retinopathy detection; significant cost-effectiveness	High accuracy, cost-effectiveness	High	The algorithm for automatic eye position recognition and image clarity needs improvement. Hospital-based results may not fully represent screening outcomes in remote areas due to potential variations in eye disease types.
8	Nusinovici et al. [12]	2020	Singapore	10,033	Cross-sectional	Multi-ethnic Asian population aged 40-80	Tree-based ML model, gradient boosting machine (GBM)	None	Relative influence of risk factors	Duration of diabetes was the highest contributor to DR risk; other significant factors identified	The utility of ML in ranking a large number of risk factors, allowing to identify the highest contributors	High	Unable to investigate the timeline pattern between risk factor exposure and its effect on the disease. Some relationships may reflect consequences of primary eye diseases rather than risk factors. Considered overall ancestral genetic background rather than individual genetic contributions. Broad definition included socioeconomic variables like job category and income.
9	Yang et al. [13]	2022	China	183	Prospective observational study	Patients with age-related cataract, cataracts and diabetes mellitus, and cataracts and high myopia	Partial least-squares discriminant analysis (PLS-DA) linear support vector machine (SVM) random forests	Laser desorption/ionization mass spectrometry is utilized for the metabolic analysis of aqueous humor samples	Diagnostic accuracy, precision, recall rates	100% accuracy, precision, and recall for ARC, CDM, CHM; AUC values of 0.994 to 1	High accuracy, precision, recall	High	High recombination rate, limited electron trapping, metal aggregation, small sample volume, and limited data reproducibility.
10	Young et al. [14]	2024	USA	743,039	Retrospective, case-controlled study	Patients with 5 leading vision conditions (age-related macular degeneration (AMD), visually significant cataract, DR, glaucoma, or ocular surface disease [OSD])	5 ML methods: generalized linear model, L1-regularized logistic regression, random forest, extreme gradient boosting (XGBoost), and J48 decision tree	Traditional diagnostic methods and ophthalmologist grading	Diagnostic accuracy, sensitivity, specificity	Model performance was compared for each pathology to select the most predictive algorithm. The AUC was assessed for all algorithms for each outcome	XGBoost demonstrated the best performance, showing a prediction accuracy 78.6% and an AUC 0.878 for visually significant cataract,	High	Limited longitudinal data, absence of physicians' notes, data representation bias, data quality issues, and models trained on EHR data may not be generalizable to the larger population

Diagnostic Accuracy and Risk Factors Analysis

Key findings point to high diagnostic accuracy, as in the study by Hecht et al. (2018) [9], which confirms the high sensitivity and specificity of ML in discriminating diabetic macular edema from other etiologies, noting ML's ability for high sensitivity. Furthermore, a study by Nusinovici et al. (2020) [12] explored the relative contributions of modifiable and non-modifiable risk factors to major eye diseases using ML. For cataracts, the study identified that age was the highest influence on the risk of nuclear and cortical cataracts, with a relative influence (RI) of around 60%. The axial length was the major individual risk factor for posterior subcapsular cataracts (PSC), with an RI of 30.8%. Besides, metabolic profiles, especially increased glucose, were significantly related to an increased risk of cortical and PSC cataracts. In particular, the present study emphasizes that various risk factors should be considered in preventing and managing cataract formation, including ocular characteristics and metabolic profiles. A study by Yang et al. (2022) developed a layered binary co-ionizers-assisted aqueous humor metabolic analysis tool that accurately detected various high-risk factors like diabetes and high myopia associated with cataract incidence. This tool gave 100% accurate, precise, and recalled rates in differentiating age-related cataracts from those with diabetes mellitus or high myopia by metabolic fingerprints in the aqueous humor. These major metabolites here correspond to glucose, phenylpyruvic acid, and urea. The area under the curve for the validation cohorts had values ranging from 0.985 to 1.000. ML algorithms, including partial least-squares discrimination analysis (PLS-DA), support vector machine (SVM), and random forests, were used to analyze the metabolic data, demonstrating the significant value of aqueous humor metabolites in detecting cataracts. A study by Young et al. [14], 2024, applied ML methods to electronic health record (EHR) data to identify and refer patients at risk from primary care physicians for eye care. This research focused on several ocular conditions, including visually significant cataracts. The measures for the prediction accuracy of the ML method, especially Extreme Gradient Boosting (XGBoost), were 78.6%, with an area under the curve of 0.878 for visually significant cataracts. If integrated into primary care, such models could improve early identification and referral of patients with cataracts for treatment, probably initiated earlier and with better outcomes.

It is noteworthy that there is a study by Crane et al. (2024) [6], which evaluated the impact of simulated cataracts on the accuracy of a convolutional neural network (CNN) in detecting diabetic retinopathy (DR) using color fundus photos. The CNN, trained on pristine images, showed high accuracy (97.0%) without simulated cataracts. However, the presence of mild, moderate, and severe simulated cataracts reduced the accuracy to 93.1%, 62.8%, and 53.5%, respectively. Sensitivity significantly decreased with increasing simulated cataract severity. It has shown the need to train AI models on images that truly represent clinical conditions in the real world. Results like these are in agreement with older studies, such as Lacombe et al. (2015), which consider the use of high-throughput Fourier-transform infrared spectroscopy combined with support vector machine leave-one-out cross-validation in distinguishing diabetic from galactosemic cataracts. Such an approach provided high sensitivity of 80-94% and specificity of 87-100% in the differential diagnosis between diabetic and galactosemic cataracts, therefore opening up new perspectives for Fourier-transform infrared spectroscopy in becoming a very rapid and inexpensive diagnostic tool.

Cataract Detection and Imaging Enhancements

In a study by Li et al. (2024), the researchers evaluated the accuracy of the Ophthalmologist Robot, an AI-based automated device for early screening of multiple blindness-causing eye diseases. This study consisted of 1578 participants, and the screening accuracy assessment involved ophthalmologists and deep learning models with images captured using an ophthalmologist robot. High accuracy in the detection of cataracts and other eye diseases made this device a potentially cost-effective screening tool in remote areas where there is limited access to ophthalmologists. On diagnostic imaging techniques, deep learning models, specifically generative models, have been promising in the detection of ophthalmology diseases by creating high-quality synthesized fundus auto-fluorescence images for age-related macular degeneration diagnosis with improved diagnostic accuracy. Results have demonstrated that this may be the potential of AI in enhancing imaging-based diagnosis related to the management of cataracts.

ML in Drug Discovery

In the paper from Gao et al. (2023), an AI-based drug discovery system was applied to identify FDA-approved drugs that might reduce the risk of extraction of cataracts in patients with diabetes. The so-called KG-Predict system prioritized the candidates for drugs by analyzing large datasets. Retrospective cohort studies were conducted with EHR data for about 800,000 patients with cataracts, associating aspirin, melatonin, ibuprofen, and acetylcysteine as drugs that showed very strong evidence of risk reduction against the extraction of a cataract in diabetic patients over periods of 5, 10, and 20 years. Moreover, work by Chen et al. (2023) [5] demonstrates that ML has potential in drug discovery and shows the path to search for new aldose reductase inhibitors (ARIs) that have opened new therapeutic opportunities for diabetic cataract treatment. Moreover, automated cataract detection and grading resulted from the innovations in AI algorithms. These developments suggest that AI has a critical role in personalizing the assessment of cataract severity and, accordingly, modeling more tailored management strategies. We point out various cutting-edge ML techniques that have been under study in the diagnosis of diabetic cataracts.

Challenges and Limitations

Results are promising, but the clinical application transition of computational models requires further analysis and significant pharmacokinetic, ethical, and practical implementation hurdles. Continuous research in the field and inter-multidisciplinary collaboration will be needed for such innovative ML technologies to reach their potential in patient care, as both the ML technologies themselves evolve and diabetic cataracts have multifactorial etiologies. The limitations of Chen et al. (2023) include a lack of experimental data, aldose reductase inhibitors, and biases due to commonly studied inhibitor classes. On their part, Crane et al. (2023) found simulating actual cataract effects and generalizing results on the broader populations challenging. Gao et al. (2023) [7] commented on the evolving set of genes that represented diabetic cataracts and, moreover, the fact that there was no detailed information about drug use in the EHR database. Hecht et al. (2019) [9] commented that combining datasets from different sources may introduce biases in the diagnosis, while the small number of mixed cases reduces diagnosis potential. Lacombe et al. (2015) [10] acknowledged that their proof-of-concept study needs to be confirmed with a larger population. Li et al. (2024) [11] mentioned improved algorithms for eye position recognition and image clarity and noted that hospital-based results might not represent screening outcomes in remote areas. Nusinovici et al. (2020) [12] could not investigate the timeline patterns between risk factor exposure and disease effects and included broad socioeconomic variables in their study. Yang et al. (2022) [13] reported high recombination rates, limited electron trapping, metal aggregation, small sample volume, and limited data reproducibility. Finally, some of the limitations, according to Young et al. (2024)[14], included the need for longitudinal data, lack of physician notes, biases of data representations, and models trained using EHR data may not generalize more broadly to the general population. Moreover, a multi-national survey among ophthalmologists reveals the general acceptance of AI as a supportive tool but more concerns regarding its application as a diagnostic tool [8]. These entail many more considerations, including the assurance of data quality control and protection of patients' privacy, in addition to a lot of challenges that must be addressed to integrate these AI technologies into clinical practice successfully. Among the observations made, this summary has underlined ideas related to potential transformation. Concretely, ML in ophthalmology is expected to develop useful clinical tools against the complexities and challenges to be faced.

Discussion

Recent advances in ML have made a significant contribution to the management of diabetic cataracts. This capability has resulted in improved diagnosis and management strategies for the denting of better patient outcomes. In this discussion, different studies have been focused on chronologically to show the changing role of machine learning in ophthalmology and the challenges faced in its integration into clinics. In 2015, Lacombe et al. [10] demonstrated the effectiveness of high-throughput Fourier-transform infrared spectroscopy associated with SVM-LOOCV in the differential diagnosis of diabetic and galactosemic cataracts. This offers very high sensitivity and specificity and fast, easy, and low-cost screening, which may be a great worth for clinical application. Accurately differentiating between these two types of cataracts can lead to more focused treatment strategies, hence enhancing patient outcomes. Hecht et al.'s study (2018) [9] is based on these findings, making it clear that the use of ML in the diagnosis of diabetic cataracts will raise the level of accuracy to a high index through improved specificity and sensitivity in edema from other conditions that discern diabetic macular edema. Moreover, Dong et al. (2017) [15] and Ran et al. (2018) [16] were able to involve a CNN, which is a class of deep learning neural networks for cataract detection and grading of its seriousness.

Nusinovici et al.(2020) [12] review the growing body of evidence on risk factors for cataracts related to age, ocular characteristics such as axial length, and metabolic profiles centered on glucose levels in the blood during lens opacification development. The findings point out the complexity of the pathophysiology of cataracts and further recommend or underscore that the risk factors may require multifaceted approaches in research and clinical practice. This proves that the application of machine learning in identifying and quantifying risk factors can open up possibilities for AI to augment the current understanding and management of cataracts. Longitudinal studies should be the future of research in this regard in order to explain the temporal relationship between these risk factors in cataract development and how they may be mitigated through prescribed interventions. In addition, Alexeeff et al. (2020) [17] demonstrated that ML models using EHR data can predict visual acuity outcomes in cataract surgery. In this case, the gradient boosting model did the best, identifying preoperative corrected distance visual acuity, age, and age-related macular degeneration as very significant predictors. This paper thus further underlines the significance of using EHR data and sophisticated machine learning techniques in improving prediction accuracy for better patient counseling and surgical planning. Later, Yamauchi et al. (2021) [18] drew attention to the role played by ML models in predicting refraction after surgery with a high degree of accuracy. The investigators demonstrated that the integration of models of machine learning-like support vector regression and neural networks into clinical practice is possible. Further, they improved accuracy, which may result in better patient outcomes and even more accurate intraocular lens (IOL) power calculations. These findings underline AI and ML's increasing role in fine-tuning surgical planning and postoperative care in cataract surgery. AI also contributes to refining the process of cataract surgical management by calculating the appropriate power of IOLs for individual patients [19]. This accurate calculation improves the results of cataract surgeries to a large extent. Notably, ML has widened its scope to aspects such as electronic health record analysis, improvement of results in clinical trials, and optimization of trial participant eligibility assessment. One such study the authors draw attention to is that of the ML models applied to electronic health records, outperforming traditional approaches for prognosis prediction [20].

Continuing this trajectory, Yang et al. (2022) [13] demonstrated the potential of using aqueous humor metabolic analysis, assisted by layered binary co-ionizers, to precisely detect cataracts with high-risk factors. This method not only distinguished between age-related cataracts, cataracts with diabetes mellitus, and cataracts with high myopia with high accuracy but also identified critical metabolites involved in these conditions. The integration of such advanced metabolic analysis tools with ML algorithms can provide a deeper understanding of cataract pathophysiology and facilitate the development of targeted interventions. This study underscores the importance of molecular-level analyses in enhancing diagnostic precision and personalizing cataract treatment strategies.

Later, in 2023, Chen et al. [5] opened avenues to a broader understanding of diabetic cataractogenesis, thus advocating for a comprehensive examination of oxidative stress, advanced glycation end-products, and metabolic disturbances for a more integrated management paradigm. Chen et al. utilized the characteristic features of all known ARI to construct a computational model that would guide them in sifting through several compounds to identify new inhibitors. This methodology connotes the power of ML and data analytics to scour through vast variables, looking for compounds with optimum characteristics for ARIs with the hope that better treatments for diabetic cataracts can be brought about. The drugs suggested by the ML model are potential treatments of cataracts for which evidence-based scientific research papers have been validated. Therefore, the call for a valid process is necessary, and strict analysis is required to ensure that the predicted drugs are safe and effective. Gao et al. [7] further illustrated the efficacy of payer-approved drugs against cataract extraction risk in diabetic patients through an AI-based drug discovery approach. These identified drugs, such as aspirin, melatonin, ibuprofen, and acetylcysteine, exhibited remarkable protective effects in diabetic subjects with cataracts. These results gave an indication that AI-aided drug repurposing can efficiently recognize new therapeutic functions of already-known drugs with the potential for delaying cataract progression and reducing the need for surgical interventions in populations affected by diabetes. More importantly, Cruz-Vega et al. [21] emphasized that well-labeled publicly available databases are among the important requirements in developing and validating machine learning algorithms in medical image analysis. The Nuclear Cataract Database allows several different machine-learning approaches and their performance related to nuclear cataract classification to be compared. In the paper, it will be shown that the proposed NCC-net outperforms them, proving its potential to assist in the pre-diagnosis or grading of a cataract and thus improve clinical decision-making processes. Conversely, the United States Food and Drug Administration Agency has published guidance to have regulations and scopes that take up the challenges that are continuously emerging as one tries to grapple with an adoption model for AI [22]. Implementing such regulations is necessary to reduce the risk of bias without affecting the development of AI models.

This was followed by the work of Young et al. [14] in 2024, which demonstrated that EHR data could be used directly for the deployment of ML models for identifying a patient at risk of visually significant cataracts. The XGBoost model used in this study has high accuracy, showing its effectiveness in real-world primary care settings. This method could greatly improve the system of triaging and referral, ensuring appropriate and timely care for cataract patients by specialists. The results reaffirm the integration of AI decision-support tools into primary care, which will have an impact on improving the early detection and clinical management of cataracts, hence reducing the burden of undiagnosed or unmanaged eye conditions. In this regard, Li et al. [11] were successful in showing that the Ophthalmologist Robot has the potential to be an effective tool for preliminary cataracts and causes-of-blindness eye disease screening. The results of this study, therefore, highlighted the ability to integrate AI-based screening tools into community and remote healthcare settings to overcome deficiency in ophthalmologists and thus better prevent early detection rates. It is envisioned that technologies, for that matter, may make a huge impact in enhancing early intervention and treatment outcomes due to the high accuracy of the Ophthalmologist Robot in cataract detection. This is because it is on the way from computational models to real-world realization into usable clinical applications, so a few challenges are involved [23]. This must be due to its pharmacokinetic and pharmacodynamic properties, together with related side effects that are possibly at the root of complications, which have to be very well investigated during the realization of an appropriate efficacy and safety in relation to the patient.

Moreover, the ability to up-scale at the individual level with respect to aspects and ethical deployment of ML technologies into healthcare settings is a task that requires rigorous validation at the level of their integration into routine clinical practice on an individual basis. Crane et al. (2024) underline AI algorithms' challenges when applied to images with real-world clinical noise as cataracts. Further reducing the accuracy and sensitivity for DR detection in the presence of simulated cataracts lends further credence to training AI models on diverse datasets that include common ocular comorbidities. This would enhance the robustness/reliability of accurate diagnosis even in severely image quality, impaired pathologies like cataracts, of AI applications in a clinical setting. It is noteworthy that simulated cataracts refer to a software-based approach in which color fundus photos are blurred by applying different magnitudes of Gaussian blur to model the degree of vision loss a person experiences with cataracts. It was performed to assess how AI algorithms for the detection of DR would be altered by image quality degradation resembling that caused by real cataracts. In that respect, Gaussian blur levels were standardized against representing mild, moderate, and severe cataract conditions at a 20/40, 20/100, and 20/200 level of visual acuity, respectively.

A question recently inserted into an international survey among ophthalmologists indicates better preparedness for integrating AI in clinical practice as an assisting tool, with approval of 88.1 percent. Of incomes, mostly in those more than 20 years old, the acceptance rate stands at 78.8 percent for using AI as clinical decision support tools and 64.5 percent for diagnostic aids. This means there is a strong inclination toward integrating AI for clinical support in ophthalmology. This survey further revealed a clear preference to accept AI applications with regard to the detection of DR over cataract cases. This unequal acceptance level may stand as evidence of the concept of reliability and clinical utility of AI-driven diagnostic tools in a myriad of ophthalmic diseases. Contrary to this, many literature studies have established the applicability of AI in cataract management. For example, Wu et al. (2019) [24] were able to achieve high accuracy using a deep learning model in diagnosing and referring cataracts depending on the slit lamp photographs.

It is known that adopting AI faces substantial hurdles in developing countries attributed to inadequate infrastructure, scarcity of data, and financial burdens [25,26]. These barriers interfere with the progress and integration of AI models in these regions, highlighting the need for comprehensive strategies to address these challenges. Moreover, Mittermaier et al. (2023) [27] and Navarro et al. (2021) [28] have laid immense emphasis that the generalization of the work's result is hampered by the potential bias or over-fitting of the data by ML models, which require diversified datasets and robust validation methods.

In the very high list of risk factors in the development of cataracts comes diabetes mellitus, whereby the disease predisposes the prevalence of cataracts to be five times that of the normal populations, further adding to the causes of cataracts as the leading causes of world visual impairment and blindness [29]. The duration of diabetes and the effectiveness of blood glucose management are paramount in determining their risk for developing diabetic cataracts [30]. The important role of AR enzyme in developing diabetic cataracts is supported by extensive genetic research on cataracts among Hong Kong Chinese patients with type 2 diabetes [31]. Moreover, the impact of AR enzyme on the progression of diabetic cataracts is further illustrated by the inverse relationship between the concentration of AR enzyme in red blood cells and the density of epithelial cells in the cataract-affected lenses of diabetic patients [32].

This paper underscores the necessity for enhancing the focus of ongoing research on interdisciplinary collaboration in order to improve and update ML models, keep pace with the rapid advancements in technology, and augment our understanding of diabetic cataract pathophysiology. Most importantly, the approach from a collaborative perspective promises to narrow the gap existing between computational potential and clinical reality in such a way that patient care can improve significantly. We acknowledge the fact that the clinical implication of the current review would largely be taken into consideration by the availability of quality data, the scalability of ML algorithms, and their interpretability in clinical settings. In reality, ML in diabetic cataract management portends improved diagnostic acuity and personalized strategies for optimal surgical outcomes. Future work to improve the applicative potential of ML methods in clinical settings will have to be centered on developing more interpretable models, ensuring the availability of large, diverse, and representative data, and seamless integration within the contemporary clinical workflow.

## Conclusions

In conclusion, implementing ML in managing diabetic cataracts represents a pivotal advancement in ophthalmology, offering a promising future for diagnosis, treatment, and optimal patient care. This review underlines ML's promises to revolutionize therapeutic approaches with them, the challenges in clinical translation, the plethora of ethical issues, and the need for constant improvement of computational models. As we navigate these complexities, multi-disciplinary collaboration will be essential in illustrating ML's full potential in clinical settings, ultimately enhancing patient outcomes in diabetic cataract management.
